# Targeting the integrated stress response in hematologic malignancies

**DOI:** 10.1186/s40164-022-00348-0

**Published:** 2022-11-08

**Authors:** Gus O. Nwosu, Jason A. Powell, Stuart M. Pitson

**Affiliations:** 1 Centre for Cancer Biology, University of South Australia and SA Pathology, UniSA Bradley Building, North Tce, Adelaide, SA 5001 Australia; 2 Adelaide Medical School, University of Adelaide, Adelaide, Australia; 3 School of Biological Sciences, University of Adelaide, Adelaide, Australia

**Keywords:** Integrated stress response, PERK, PKR, GCN2, HRI, ATF4, Hematological malignancy, Targeted therapy

## Abstract

While numerous targeted therapies have been recently adopted to improve the treatment of hematologic malignancies, acquired or intrinsic resistance poses a significant obstacle to their efficacy. Thus, there is increasing need to identify novel, targetable pathways to further improve therapy for these diseases. The integrated stress response is a signaling pathway activated in cancer cells in response to both dysregulated growth and metabolism, and also following exposure to many therapies that appears one such targetable pathway for improved treatment of these diseases. In this review, we discuss the role of the integrated stress response in the biology of hematologic malignancies, its critical involvement in the mechanism of action of targeted therapies, and as a target for pharmacologic modulation as a novel strategy for the treatment of hematologic malignancies.

## Introduction

Progress in genetic analyses have advanced our understanding of the molecular drivers underpinning hematologic malignancies, thus prompting the development of an array of targeted therapies that are revolutionizing the clinical management of these diseases [1]. Targeted therapies exploit molecular vulnerabilities unique to cancer cells and typically alter cellular signaling pathways to inhibit tumorigenic growth and promote cell death. However, despite successful implementation of these agents in the treatment of hematologic malignancies, intrinsic and acquired resistance remains an ongoing challenge [1]. Therefore, an improved understanding of the molecular signaling pathways modulated by targeted therapies is critical to enhancing the efficacy of these agents.

The integrated stress response (ISR) is an adaptive signaling pathway that contributes to the biology of a number of hematologic malignancies since they are inherently prone to numerous cellular stresses [2]. Furthermore, growing evidence has found the ISR to be involved in the activity of a number of targeted therapies used in the treatment of hematologic malignancies; thus, indicating the therapeutic potential of ISR modulation as a strategy for improving the efficacy of targeted therapies. Here, we provide an overview of the ISR and the role the various ISR pathway components play in hematologic malignancies. We also discuss the evidence for the emerging role of the ISR in mediating the efficacy of targeted therapies and the significance of these findings for developing new therapeutic approaches for hematologic malignancies.

### Overview of the integrated stress response

**Fig. 1:**
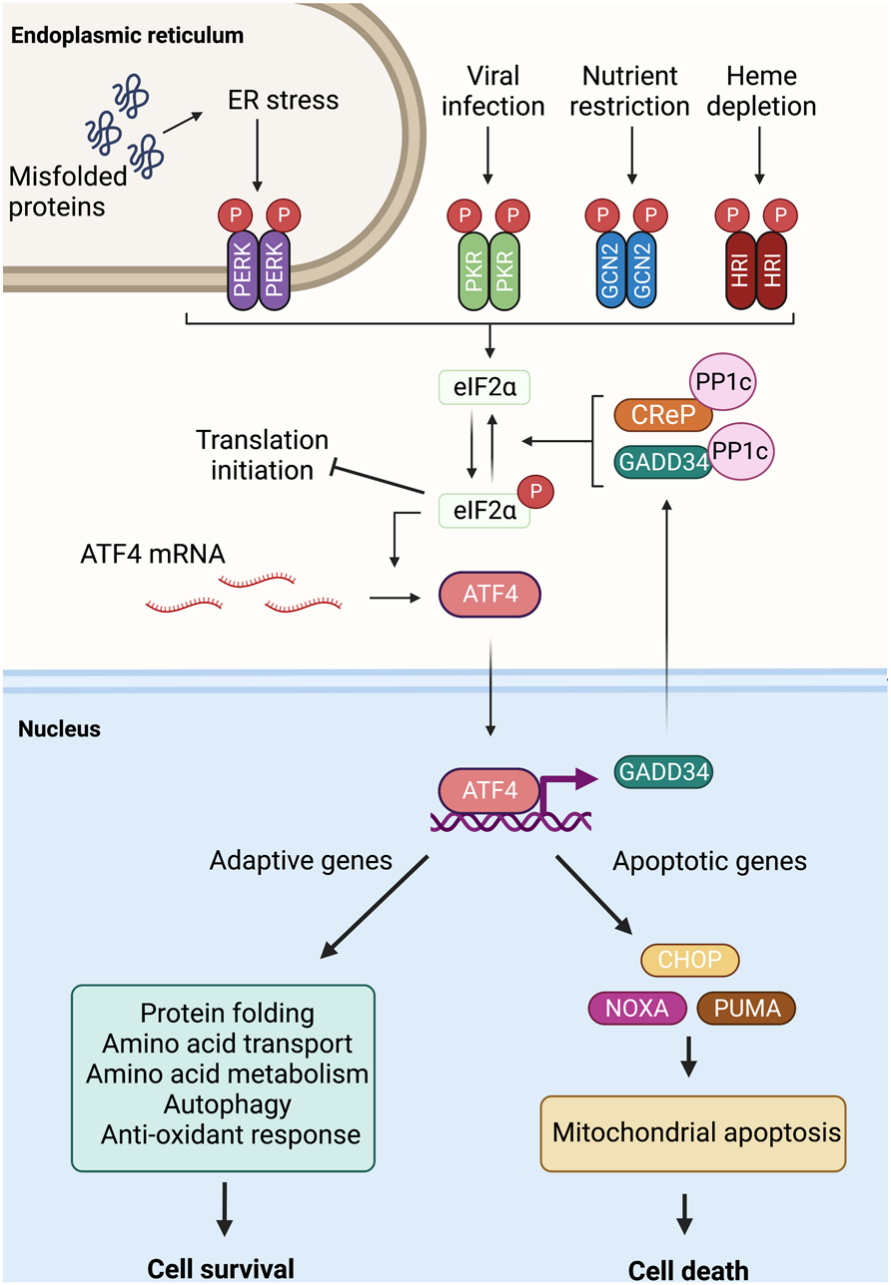
Overview of the integrated stress response pathway. Distinct cellular stresses cause the phosphorylation and activation the eIF2α kinases PERK, PKR, GCN2 and HRI. The eIF2α kinases phosphorylate Ser51 of eIF2α which leads to suppression of global protein synthesis but selective enhancement of translation of some mRNAs, such as that encoding ATF4. ATF4 is a transcription factor that activates genes which regulate cellular responses to stress signals to determine cell fate. Additionally, in a negative feedback loop, ATF4 drives the expression of GADD34, a protein phosphatase 1 (PP1) regulatory subunit which forms a complex with the PP1 catalytic subunit (PP1c) to dephosphorylate and inactivate eIF2α. Another PP1 regulatory subunit, CReP, is constitutively expressed independent of ISR signaling and functions to maintain basal levels of de-phosphorylated eIF2α in the absence of stress. Created with BioRender.com

The ISR (Fig. 1) is a complex signaling pathway that regulates cellular responses to stress stimuli and enables either adaptation or the instigation of cell death mechanisms [2]. Stress signals are transmitted through the ISR via four serine/threonine kinases PERK, PKR, GCN2 and HRI which in response to different forms of stress phosphorylate a shared target, eukaryotic initiation factor 2α (eIF2α); hence they are collectively known as the eIF2α kinases [4]. Phosphorylation of eIF2α on Ser51 inhibits 5’ cap-dependent mRNA translation, resulting in the global suppression of protein synthesis to facilitate adaptation to a variety of stresses linked to protein synthesis, including proteotoxic stress, viral replication, heme depletion and amino acid withdrawal [2]. Paradoxically, eIF2α phosphorylation also results in the increased translation of select mRNA bearing upstream open reading frames that favor 5’ cap-independent translation [3]. This selective translation results in the synthesis of activating transcription factor 4 (ATF4) which modulates gene expression to coordinate stress responses, including regulation of amino acid and protein homeostasis, autophagy, anti-oxidant responses and mitochondrial apoptosis all of which ultimately influence cell fate [2, 4]. Activation of the ISR via eIF2α phosphorylation is also regulated by phosphatases which act either constitutively to maintain basal eIF2α dephosphorylation [5] or in response to ISR activation as negative feedback regulation [6].

These mechanisms regulating eIF2α phosphorylation underscore the importance of controlled ISR activity since in addition to regulating stress adaptation, prolonged or intense stimulation of the ISR can also result in ATF4-driven expression of pro-apoptotic effectors to induce cell death [2]. Thus, the components of the ISR facilitate the tight regulation of rapid and diverse responses to a range of stress stimuli to either reinstate cellular homeostasis and promote cell survival or, if this is not possible, trigger apoptotic cell death.

### The eIF2α kinases

#### PERK

**Fig. 2:**
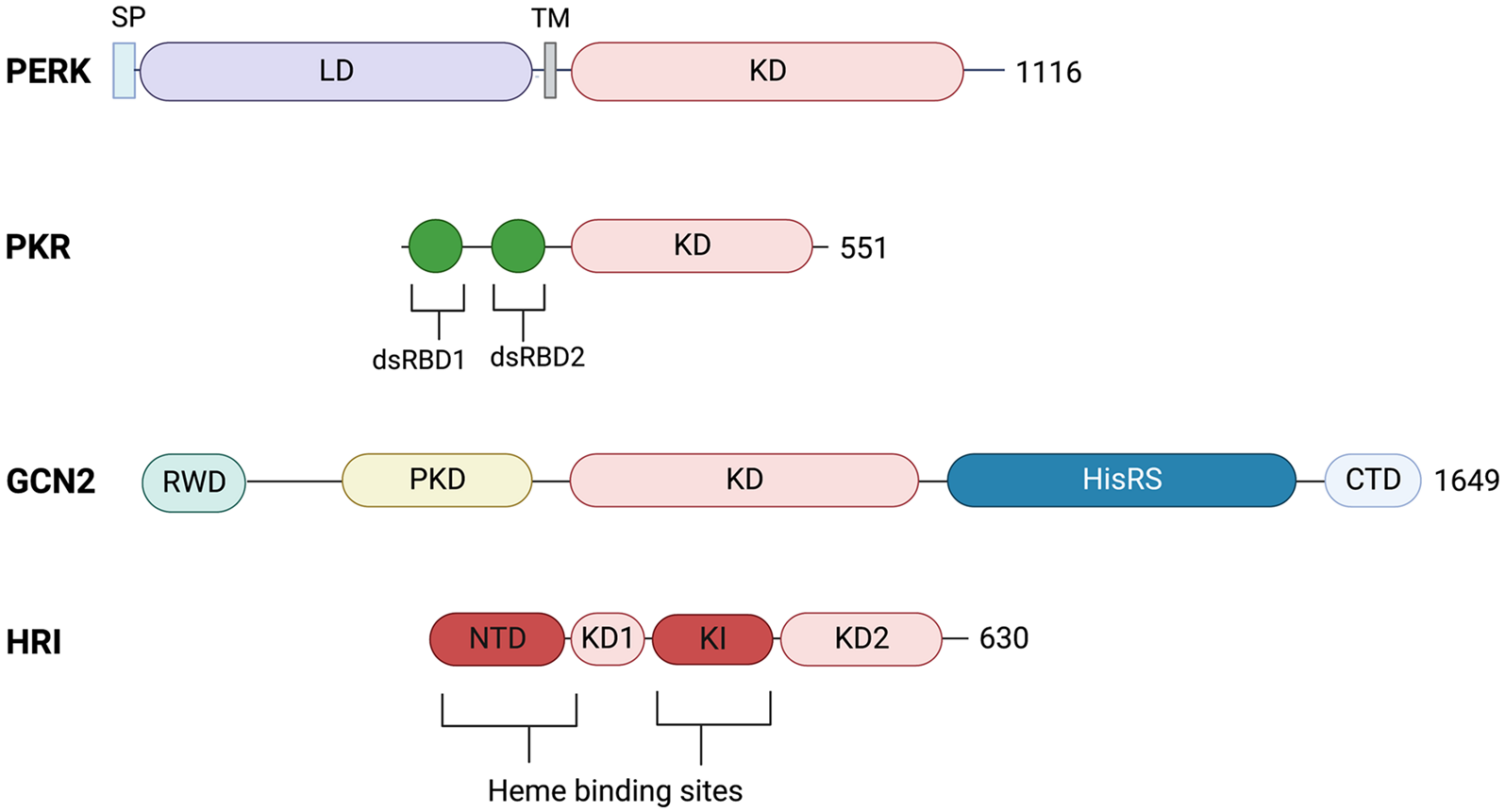
Domain structures of the eIF2α kinases. Structures are oriented from N termini to C termini following left to right. Total amino acid number is listed at the C terminal ends. Abbreviations: signal peptide (SP), luminal domain (LD), kinase domain (KD), double stranded RNA binding domains (dsRBD), pseudokinase domain (PKD), histidyl-tRNA synthetase (HisRS) domain, C-terminal ribosome binding/dimerization (CTD) domain, N-terminal heme binding (NTD) domain, kinase domain 1 (KD1), kinase insert (KI) domain, kinase domain 2 (KD2). Created with BioRender.com

**Fig. 3:**
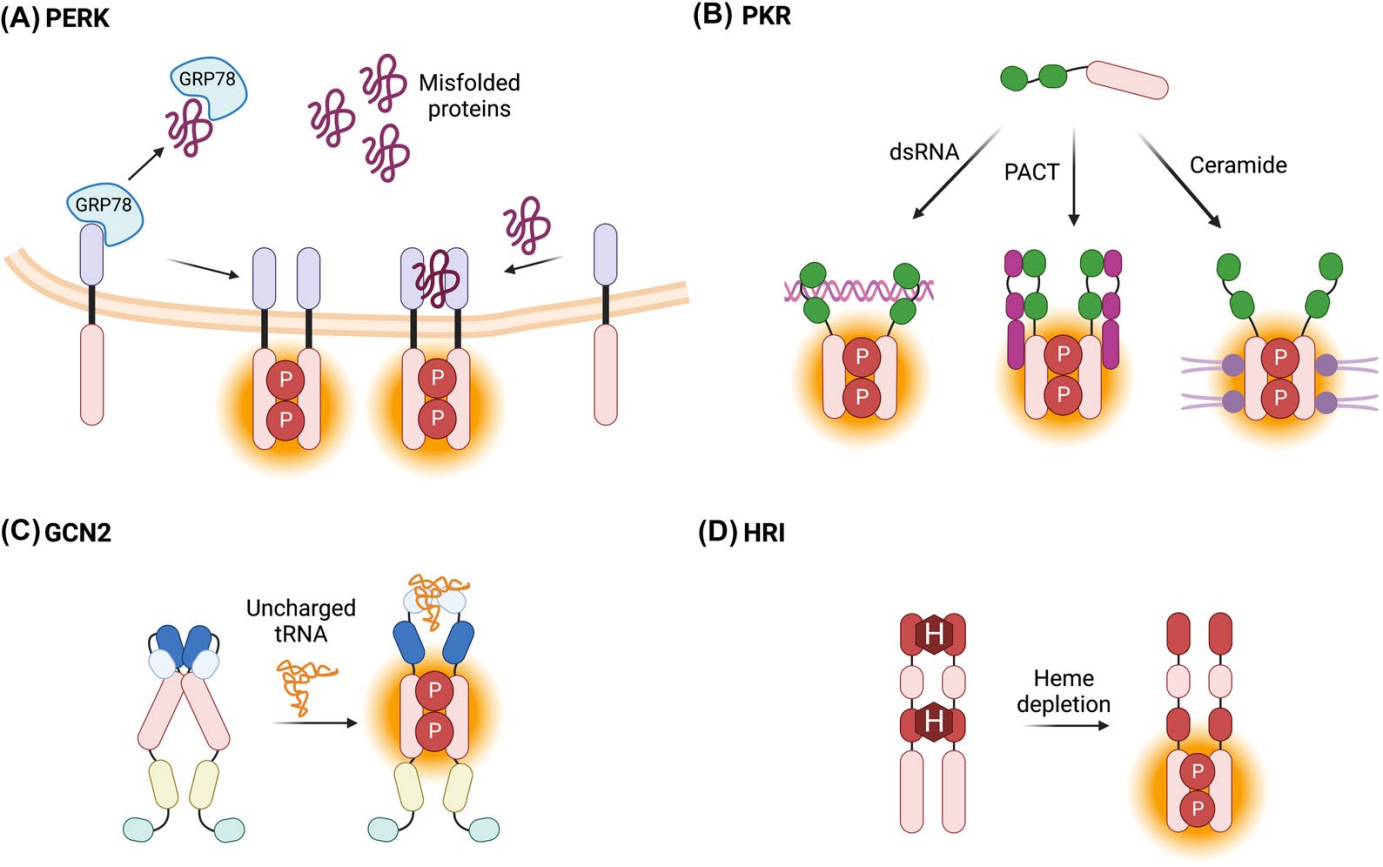
Activation mechanisms of the eIF2α kinases. Diverse stimuli cause the eIF2α kinases to undergo activation via autophosphorylation (indicated by orange shading of kinase domains). **A** In the absence of ER stress GRP78 binds to the luminal domain (purple) of PERK preventing it from dimerizing and undergoing activation via trans-autophosphorylation (P). The presence of misfolded proteins titrates GRP78 from PERK which facilitates dimerization converting PERK into an active kinase (activation status shown in orange). Misfolded proteins can also interact directly with the luminal domain to stimulate PERK activation. **B** PKR is canonically activated by interaction between the double stranded RNA binding domains (dsRBD, shown in green) and dsRNA. PKR can also be activated by direct interaction with the PKR activating protein PACT (shown in pink). The lipid ceramide (purple) can also directly interact with PKR to stimulate its activity. **C** During nutrient availability, GCN2 is an inactive homodimer due to inhibitory interactions between the C-terminal domain (CTD), histidyl-tRNA synthetase (HisRS) domain and kinase domains. Amino acid depletion causes an increase in levels of uncharged tRNA which displace the CTD causing rearrangement of the regulatory regions to facilitate activation of the enzyme. **D** Heme (H) binds to the HRI homodimer to stabilize it in an inactive conformation. Depletion of heme allows HRI to adopt an active configuration. Created with BioRender.com

In contrast to the other eIF2α kinases which are mainly cytosolic, PERK (protein kinase R-like endoplasmic reticulum kinase) is localized to the endoplasmic reticulum (ER) membrane, containing an N-terminal ER luminal domain linked to a C-terminal cytosolic kinase domain [7] (Fig. 2). PERK is also unique amongst the eIF2α kinases in being a member of the ER stress/unfolded response pathway [8]. Indeed, the canonical function of PERK is to detect the accumulation of misfolded proteins in the ER lumen (ER stress) and, thus, activated PERK phosphorylates eIF2α to suppress translation and protect cells from further build-up of misfolded proteins in the ER [7]. Sustained or intense PERK activation, however, has also been shown to suppress cell growth and stimulate apoptosis [8], highlighting that PERK elicits dichotomous effects on cell fate in a context dependent manner. In the absence of ER stress, PERK is negatively regulated by association with GRP78 (78 kDa glucose-regulated protein; also known as BiP), an ER lumen resident member of the heat shock protein family of molecular chaperones [7]. Upon ER stress, GRP78 dissociates from PERK and binds to misfolded proteins which in turn permits PERK monomers to dimerize via their luminal domains, resulting in autophosphorylation at Thr980 and activation of the enzyme [7] (Fig. 3A). There is also evidence supporting non-canonical PERK activation whereby dimerization of PERK is driven by direct interaction between misfolded proteins and the PERK luminal domain [9] (Fig. 3A).

PERK, encoded by the *EIF2AK3* gene, is expressed ubiquitously in normal tissues (albeit at higher levels in secretory tissues such a pancreatic islet cells) [10] and is also present and active in hematologic malignancies, including multiple myeloma (MM) [11] and chronic myeloid leukemia (CML) [12]. MM is a malignancy characterized by the overproduction of paraprotein by monoclonal plasma cells which results in increased ER stress [13]. Unsurprisingly, studies of MM cells showed that molecular knockdown of PERK causes cell death [11], thus highlighting the important pro-survival function of PERK in this disease. ER stress has also been observed in CML and has been shown to occur as a result of the activity of the Bcr-Abl oncogene characteristic of this disease [12]. As a consequence of this, CML cell lines and leukemic stem cells from patient samples exhibit elevated PERK and phospho-eIF2α protein levels compared to healthy controls and expression of both is positively correlated with more advanced disease stage [12]. Increased PERK signaling and phospho-eIF2α levels have also been observed in primary lymphoma cells and were attributed to c-Myc driven activation of protein synthesis and misfolded protein accumulation [14]. Recent analyses of pan-cancer PERK expression have also identified significant downregulation of PERK mRNA in a number of lymphoma sub-types [15]; however, the functional significance of this finding has yet to be examined.

#### PKR

Protein kinase R (PKR) is a mainly cytosolic enzyme encoded by the *EIF2AK2* gene. The 68 kDa protein consists of two separate N-terminal double-stranded RNA (dsRNA) binding domains connected to a bi-lobed C-terminal kinase domain by a flexible linker region of approximately 80 amino acids [7] (Fig. 2). PKR is ubiquitously expressed at basal levels but can also be induced by type I interferons [7]. Although PKR is best characterized as a cytoplasmic protein, nuclear localization of PKR has also been reported, possibly due to post-translational modifications [16]. Activation of PKR typically occurs in response to viral dsRNA [7] and involves binding of dsRNA to the dsRNA binding domains that induces homodimerization of PKR monomers and trans-autophosphorylation of the enzyme on Thr446 (Fig. 3B) which triggers eIF2α-mediated suppression of translation to inhibit the synthesis of viral proteins [17]. However, PKR activity can also be stimulated by endogenous dsRNAs [18], nutrient excess, ER stress [19], oxidative stress [20], the lipid ceramide [21] and the protein PACT which in response to stress stimuli binds to PKR to promote autophosphorylation and activation of the kinase [20] (Fig. 3B).

In addition to phosphorylating eIF2α, PKR also functions as an activator of NF-κB, and as a mediator for toll-like receptor signaling and inflammasome activation [22]. Thus, the diverse signaling functions of PKR prompted its examination in disease and has led to the discovery of its roles in hematologic malignancies. For instance, early studies showed that PKR expression is increased in both acute lymphoblastic leukemia (ALL) and acute myeloid leukemia (AML) patient samples, with significantly higher PKR mRNA found in relapsed AML samples compared to samples obtained at diagnosis [23]. These findings pointed to a functional role for PKR in these malignancies and were further supported by analyses of PKR protein levels in AML patient samples which revealed that high PKR expression correlated with reduced overall survival and shorter disease remissions [24]. Subsequent studies showed that in addition to elevated expression, AML and ALL cell lines also exhibited enrichment of activated PKR, and selective inhibition of this enzyme reduced the growth and viability of these cells [25]. Additionally, bone marrow failure which is characteristic of hematopoietic disorders such as myelodysplastic syndrome (MDS) and subsequent progression to AML has also been shown to involve the activity of PKR. Murine transgenic studies found that PKR expression resulted in dysplastic changes to hematopoietic tissues, moderate cytopenia, and the production of myeloid and lymphoid blasts characteristic of bone marrow failure [26]. Interestingly, an analysis of bone marrow tissue from MDS patients at high-risk of progressing to AML showed increased activation and nuclear localization of PKR [27]. Subsequent studies in a murine model of MDS/leukemia revealed that nuclear PKR enhanced MDS progression to leukemia by inhibiting DNA damage repair mechanisms and promoting the acquisition of somatic mutations [24].

Despite these reported tumor supporting properties of PKR, recent studies have shown that drug-induced PKR activation has anti-tumor effects, causing ATF4-stimulated expression of the pro-apoptotic protein NOXA, resulting in AML cell death [21]. However, PKR has a well-established dual function as both a pro-survival and pro-apoptotic mediator, a role which varies depending on the amplitude and type of stimulus [17]. Thus, this divergence in PKR function is likely due to differences in the signaling, whereby a small amount of PKR activity induced by endogenous chronic stressors in cancer cells is pro-survival/pro-proliferative, whereas the induction of further PKR activity by exogenous acute stimuli leads to apoptosis. Therefore, PKR plays diverse roles in the pathogenesis, survival, and evolution of hematologic malignancies.

#### GCN2

GCN2 (general control non-derepressible kinase 2), encoded by the *EIF2AK4* gene, is the largest of the four eIF2α kinases (190 kDa) and contains the most regulatory domains [28] (Fig. 2). Flanking the kinase domain, GCN2 possess a N-terminal RWD domain (so called for its occurrence in Ring-finger proteins, WD-repeat containing proteins and DEAD-like helicases), a pseudokinase domain, as well as a histidyl-tRNA synthetase (HisRS) domain and a C-terminal ribosome binding/dimerization (CTD) domain [28]. GCN2 is activated in response to amino acid deprivation [28]. Thus, during nutrient availability, auto-inhibitory interactions between the HisRS and CTD domains with the kinase domain maintain GCN2 in an inactive conformation [28]. Amino acid depletion leads to elevated levels of uncharged tRNA molecules in the cell which disrupt GCN2 auto-inhibition by binding to the HisRS domain causing it to dissociate from the CTD domain. Upon loss of auto-inhibition, the GCN2 dimer undergoes conformational restructuring resulting in exposure of the catalytic cleft and ensuing autophosphorylation at Thr882 and Thr887 and activation of the enzyme [28] (Fig. 3C). Activated GCN2 phosphorylates eIF2α to suppress protein synthesis, thus alleviating the impact of nutrient depletion and lowering demand for amino acids while also stimulating the upregulation of amino acid transport genes [28]. In addition to amino acid depletion, GCN2 has also been shown to be activated by glucose restriction (resulting in depletion of cytosolic amino acids), UV radiation, oxidative stress [29] and in response to stress-induced ribosome collisions [30].

Hematologic malignancies display increased demand for nutrients and exhibit dysregulated metabolic signaling in order to attain amino acids for their continued survival [31]. Thus, studies have shown that amino acid depletion activates GCN2 in ALL cell lines and inhibition of the enzyme sensitizes both ALL and AML cell lines to treatment with L-asparaginase [32], a first-line therapy for ALL and prospective therapy for AML [33]. Recent single-cell transcriptomic analyses have also described a role for GCN2 in the pathogenesis of MM with the finding that in a murine model of MM progression, the GCN2 signaling pathway was enriched in disease cells with the greatest enrichment seen during the early stage of the disease [34]. Furthermore, targeting GCN2 in MM cell lines caused apoptosis and reduced cell viability, thus indicating a role for GCN2 signaling in MM, possibly as an adaptive mechanism to cope with amino acid depletion caused by elevated immunoglobulin production in these cells [34]. Taken together these findings point towards an important role for GCN2 in the biology of hematologic malignancies that warrants further investigation.

#### HRI

The heme regulated inhibitor (HRI) protein is primarily known for its role in regulating the synthesis of globin proteins in accordance with the availability of heme [35] whereby it phosphorylates eIF2α to suppress protein synthesis and prevent the proteotoxicity associated with the aggregation of globin proteins [36]. HRI contains two distinct heme-binding sites, an N-terminal heme binding domain (NTD) and a unique heme binding kinase insertion (KI) domain that bifurcates the C-terminal kinase domain (KD1 and KD2) [7] (Fig. 2). While heme appears constitutively bound to the NTD, heme-mediated regulation of HRI is brought about through a concentration-dependent association of heme with the KI domain [7]. During normal homeostatic conditions, heme binds to the KI domain causing HRI to adopt an inactive dimeric conformation [7]. Binding of heme to the KI domain is reversible and during heme deficiency, loss of heme binding permits an active conformation of the HRI dimer which then undergoes autophosphorylation at Thr485, resulting in activation of the enzyme [7] (Fig. 3D). Activation of HRI can also occur in response to arsenite and heat-shock through as yet undefined mechanisms [37], and by mitochondrial stress whereby the mitochondrial protein DELE1 is cleaved and released into the cytoplasm and interacts directly with HRI to stimulate its activity [38].

Expression of HRI was previously thought to be restricted to erythroid cells [36], however, recent findings suggest that HRI may be expressed more broadly, including in some hematologic malignancies such as MM and Ph^+^ B-cell ALL (B-ALL), where HRI activity has been implicated in the regulation of apoptosis [39, 40]. Indeed, in MM, pharmacological activation of HRI led to increased phospho-eIF2α and caused cell death in both cell lines and patient samples [39]. Similarly, pharmacological activation of HRI in B-ALL cell lines resulted in a suppression of the pro-survival protein MCL1 (a critical effector of the mitochondrial apoptotic pathway) to sensitize B-ALL cells to the activity of BH3-mimetics (potent inducers of apoptosis) [40]. Notably, this effect was abrogated by genetic ablation of the HRI gene *EIF2AK1* [40], thus underscoring the important role of HRI in the regulation of apoptosis in B-ALL. Ultimately, the function of HRI in hematologic malignancies remains poorly understood but growing evidence suggests HRI may be a critical mediator in determining cancer cell fate.

### Regulation of eIF2α

**Fig. 4:**
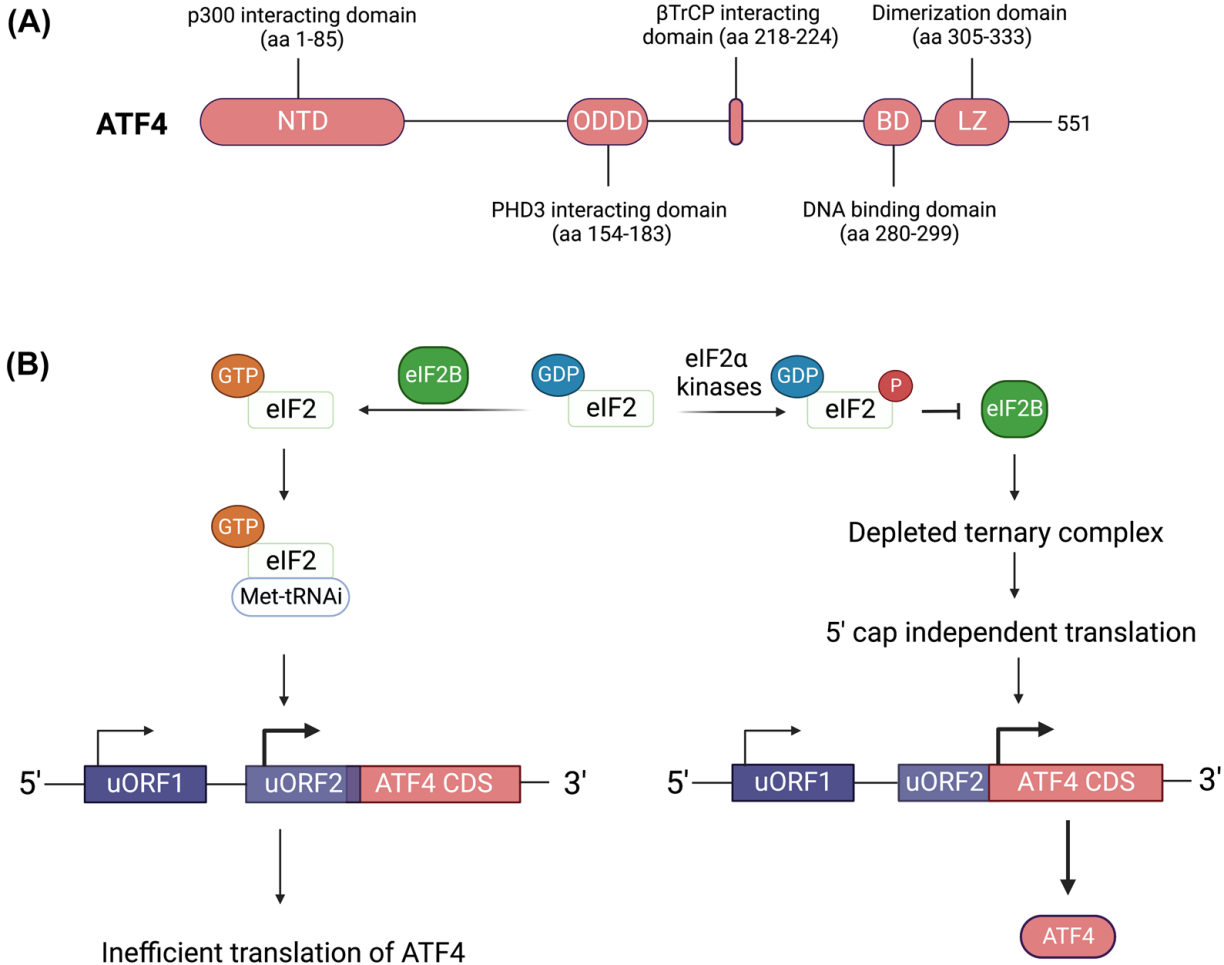
ATF4 protein structure and translational regulation by eIF2α phosphorylation. **A** The ATF4 protein consists of several of regulatory and functional domains. The regulatory domains include the N-terminal domain (NTD), oxygen dependent degradation domain (ODDD) and β-transducin repeat-containing protein (βTrCP) interacting domain all of which regulate the stability of ATF4 in response to interactions with histone acetyltransferase p300, prolyl-4-hydroxylase 3 (PHD3) and βTrCP respectively. The functional domains include a basic domain (BD) involved in DNA binding and a leucine zipper (LZ) domain that facilitates dimerization. **B** Under normal conditions eIF2B catalyzes the transfer of eIF2-bound GDP (inactive) for GTP to produce the eIF2-GTP (active) that forms a ternary complex with Met-tRNA_i_. In the presence of sufficient ternary complex 5’ cap dependent translation proceeds resulting in inefficient translation of ATF4 mRNA due to the presence of inhibitory upstream open reading frames (uORF) in the 5’ untranslated region. Presence of cellular stress causes eIF2α kinases to phosphorylate eIF2 causing it to act as an inhibitor of eIF2B thus preventing further regeneration of eIF2-GTP. This leads to the depletion of the ternary complex which favors ribosome re-initiation at the ATF4 coding sequence (CDS) and enhanced synthesis of ATF4 protein. Created with BioRender.com

The eIF2 protein complex is a key modulator of protein synthesis that is regulated by eIF2α kinases [41]. The eIF2 protein is a tri-partite complex consisting of α, β and γ subunits and is involved in facilitating the initiation of translation whereby it forms a ternary complex (TC) with GTP and methionyl-initiator tRNA (Met-tRNA_i_) [42]. The TC binds to the 40S ribosomal subunit to form the pre-initiation complex (PIC) which then associates with the 5’ cap of mRNA [41]. Upon binding the 5’ cap, the PIC scans the 5’ UTR region of the mRNA until pairing between Met-tRNA_i_ occurs at which point GTP on the ternary complex is hydrolyzed to GDP with subsequent binding of the 60S subunit, release of eIF2-GDP and initiation of translation [41]. In order to replenish active eIF2, the guanine nucleotide exchange factor eIF2B binds to the eIF2 complex and catalyzes the exchange of GDP for GTP, thus facilitating continued initiation of translation (Fig. 4) [42].

In the presence of cellular stress eIF2α kinases phosphorylate the α subunit of eIF2 on Ser51 which causes the eIF2 complex to bind to and inhibit the function of eIF2B, thus preventing the regeneration of eIF2-GTP, lowering the concentration of TC and ultimately inhibiting the initiation of 5′ cap-dependent translation [43]. However, under these conditions, and in specific contexts, the 5’cap-independent translation of select mRNA with upstream open reading frames is favored resulting in the selective synthesis of key proteins such as ATF4, ATF5 and CHOP, all of which are involved in regulating cellular responses to stress [4]. Through this mechanism, stress signals detected by the eIF2α kinases initiate a dual response whereby (1) translation is globally suppressed to alleviate stress arising from protein synthesis/nutrient depletion and (2) stress responsive genes are upregulated to coordinate an adaptive response.

Upon resolution of cellular stress, integrated stress response signaling is attenuated by the dephosphorylation of eIF2α. eIF2α is dephosphorylated by protein phosphatase 1 complexes containing either the CreP [5] or GADD34 [6] regulatory subunits (Fig. 1). These phosphatase complexes regulate eIF2α in different contexts, with CreP operating constitutively in the absence of stress to maintain low basal levels of eIF2α activity [5], whereas GADD34 expression is upregulated in response to stress and functions as a negative feedback inhibitor of ISR signaling [6].

Thus, eIF2α exists at the nexus of control of the ISR and is subject to tight regulation by the eIF2α kinases and phosphatases. Furthermore, since phosphorylation of eIF2α is the critical step in the transduction of stress signals detected by the eIF2α kinases, it is therefore central to ISR signaling observed in hematologic malignancies. Beyond this role in mediating the ISR, additional functions of eIF2α in the pathogenesis of hematologic malignancies have been described. For instance, CML cell lines ectopically expressing an eIF2α Ser51 → Ala mutant (blocking phosphorylation at this site and eIF2α activation) had reduced expression and secretion of matrix metalloproteinases (MMPs), enzymes which are involved in the degradation of extracellular matrix components [44]. This finding was accompanied by the observation that loss of eIF2α activity reduced the invasiveness of CML cells. This, together with observations that conditioned media from these cells led to similar reductions in the invasiveness of bone marrow stromal fibroblasts, implicates eIF2α in regulating the bone marrow remodeling and invasive potential of CML cells [44]. These data are in agreement with the finding that primary CML cells also exhibit increased phospho-eIF2α compared to healthy donors, with phospho-eIF2α substantially increasing in cells from patients at the advanced blast crisis stage of the disease [12]. Thus, eIF2α appears to be involved in the disease progression of CML.

### ATF4: the master regulator of the ISR

ATF4 (activating transcription factor 4; also known as CREB-2) is part of the basic leucine zipper (bZIP) family of transcription factors and is the key effector mediating the adaptive responses stimulated by the ISR [45]. Reflecting its broad role in the coordination of gene expression in response to stress stimuli, ATF4 is subject to regulation at the transcriptional, translational and post-translational levels [2]. Under normal conditions, ATF4 mRNA is basally expressed at low levels however, stress stimuli induce an increase in ATF4 transcription [46, 47]. The epigenetic modulator protein arginine methyltransferase 5 (PRMT5) is also involved in the regulation of ATF4 mRNA splicing and stability [48]. However, ATF4 expression is primarily regulated at the translational level, via stress signaling integrated through eIF2α which stimulates upregulated translation of ATF4 mRNA. The mechanism for this regulation (Fig. 4) is based on the unique properties of the ATF4 mRNA which contains two upstream open reading frames (uORF) in the 5’ UTR region prior to the coding sequence [49, 50]. Under normal conditions, ATF4 mRNA is inefficiently translated since ribosomes initiate translation at uORF1 and rapidly re-initiate at uORF2 which extends into the ATF4 coding sequence [49, 50]. Phosphorylation of eIF2α leads to reduced levels of eIF2-GTP-Met-tRNA_i_ which causes delayed ribosomal re-initiation and favors selective re-initiation at the ATF4 coding sequence. Given that ATF4 mRNA is constitutively expressed at basal levels, this translational mechanism allows for the rapid coordination of adaptive responses and bypasses the eIF2α-meditated suppression of translation.

Structurally, the ATF4 protein contains a number of functional and regulatory domains, summarized in Fig. 4, including a N-terminal domain for binding the histone acetyltransferase p300 [51], an oxygen dependent degradation domain (ODDD) for binding with prolyl-4-hydroxylase domain 3 (PHD3) [52], a domain for interacting with the SCF E3 ubiquitin ligase component β-transducin repeat-containing protein (βTrCP) and a C-terminal leucine zipper region that facilitates dimerization [53]. Binding of p300 to ATF4 enhances its stability and transcriptional activity [51]. Likewise, binding of PHD3 also enhances ATF4 stability [4]. In contrast, binding of βTrCP results in the ubiquitination and degradation of ATF4 [54]. ATF4 is also subject to phosphorylation, acetylation, and methylation as well as interactions with non-bZIP family proteins, all of which either alter ATF4 activity or target gene selectivity [2, 4]. The bZIP transcription factors typically homodimerize or heterodimerize (via the bZIP domain) and ATF4 has been shown to primarily heterodimerize as it forms unstable homodimers in vitro [55]. Binding partners of ATF4 include CCAAT box/enhancer binding protein β (C/EBPβ), C/EBPγ, and C/EBP homologous protein (CHOP), and these heterodimers differentially influence the outcome of ATF4 target gene regulation whereby ATF4-CHOP heterodimers are associated with pro-apoptotic signaling, whereas ATF4-C/EBPβ and ATF4-C/EBPγ heterodimers regulate adaptation to stress [4]. Functionally, ATF4 behaves as both a transcriptional repressor and transcriptional activator [2]. Key ISR target genes of ATF4 include CHOP, GADD34 and CReP (negative regulators of eIF2α) [56]; however, ATF4 also has a wide distribution of target genes that regulate diverse stress responses including apoptosis, amino acid metabolism, anti-viral responses and protein folding [4].

Cancer cells are subject to substantial cellular stress due to their dysregulated growth and metabolism and thus frequently rely on ATF4-mediated regulation of stress responses [57]. In diffuse large b-cell lymphoma (DLBCL), ATF4 knockdown was shown to substantially reduce cell proliferation, and it was postulated that ATF4 may function to ameliorate amino acid depletion in these cells caused by the flow of cytosolic amino acids into the TCA cycle [58]. AML cells have been found to be reliant on ATF4 to regulate survival upon exposure to reactive oxygen species (ROS) as it was shown that in AML cells bearing the inv (3) (RPN1-EVI1) chromosomal rearrangement, pharmacological inhibition of PRMT5, which as described above regulates ATF4 mRNA splicing and stability, led to production of an unstable, intron-retained ATF4 mRNA that was restricted to the nucleus and resulted in reduced ATF4 protein levels and increased ROS [48]. Interestingly, it was also shown that RPN1-EVI1 AML cells exhibit enhanced sensitivity to PRMT5 inhibition and likewise overexpression of EVI1 induced the degradation of the ATF4 protein and enhanced ROS, suggesting that PRMT5-mediated regulation of ATF4 is critical to counteract the deleterious oncogenic signaling of EVI1 [48].

A role for ATF4 has also been shown in the pathogenesis of Fms-like tyrosine kinase 3-internal tandem duplication (FLT3-ITD) AML as it was shown that the mutant FLT3-ITD protein stimulates ATF4-dependent autophagy to support the proliferation of AML cells [59]. Further studies have also shown that in daunorubicin resistant AML cell lines, ATF4 binding is enriched at a stress-responsive enhancer for the *ABCB1* gene which encodes the p-glycoprotein drug efflux pump implicated in multi-drug resistance; thus, indicating a potential role for ATF4 in mediating drug-resistance in AML [60]. Additionally, primary AML leukemic stem cells exhibit increased ATF4 activity and target gene expression (61). In AML, ATF4 transcription has also been shown to be regulated by the RUNX1 transcription factor [62], which is frequently mutated in AML [1]. Interestingly, germline mutations resulting in defective RUNX1 activity led to reduced ATF4 expression and increased resistance to ER stress [62]. These findings suggest that RUNX1 mutations may confer a survival advantage by blunting pro-apoptotic signaling by ATF4 which contrasts with the canonical adaptive roles described for ATF4, but is yet indicative of its varied functions. Thus, ATF4 plays a central role in the pathogenesis of a number of hematologic malignancies.

### ISR in the therapy of hematologic malignancies

From the studies described above, it is clear that the ISR contributes to the pathogenesis of a number of hematologic malignancies. However, investigation into the mechanisms of action of targeted therapies in hematologic malignancies has revealed that the ISR also has a substantial involvement in the anti-neoplastic activity of numerous agents (Table 1); emphasizing the dichotomous signaling of the ISR. Here we summarize evidence for the role of the ISR in a number of targeted therapies currently in the clinic, or in late-stage development for treatment of hematologic malignancies.

**Table 1:** Targeted therapies that elicit ISR signaling in hematologic malignancies

Therapeutic agent	Drug class	Mode of action	Effect on ISR	Disease outcome	References
5′-azacitidine	Hypomethylating agent	Inhibition of DNA methyltransferase activity	↑ eIF2α phosphorylation	Noxa mediated apoptosis in AML	[68]
ONC201	Imipridone	Dopamine receptor D2 antagonist	↑ ATF4 translation	Apoptosis in AML and MCL cell lines ↓ MCL-1 protein expression in MCL cell lines	[74]
Pyrvinium pamoate	Anthelmintic	Inhibition of mitochondrial respiratory complex I	↑ eIF2α phosphorylation ↑ ATF4 translation	Apoptosis in FLT3-ITD AML cell lines	[80]
Atovaquone	Anthelmintic	Inhibition of mitochondrial respiratory complex III	↑ eIF2α phosphorylation ↑ ATF4 translation ↑ ATF4 target gene transcription	Apoptosis in AML cell lines and primary samples ↓ Disease burden in murine AML and MM models	[79, 83]
Bortezomib	Proteasome inhibitor	Reversible inhibitor of the 26S proteasome	↑ eIF2α phosphorylation ↑ ATF4 translation ↑ CHOP translation	↓ MM cell line viability ↑ Pro-survival response to bortezomib	[84, 87]
Carflizomib	Proteasome inhibitor	Irreversible inhibitor of the 20S proteasome	↑ ATF4 translation	↓ MM cell line viability	[86]
Marizomib	Proteasome inhibitor	Irreversible inhibitor of the 20S proteasome	↑ eIF2α phosphorylation and CHOP translation when combined with bortezomib	↓ MM cell line viability	[85]

*AML* Acute myeloid leukemia, *MM* Multiple myeloma, *MCL* Mantle cell lymphoma

### Hypomethylating agents

**Fig. 5:**
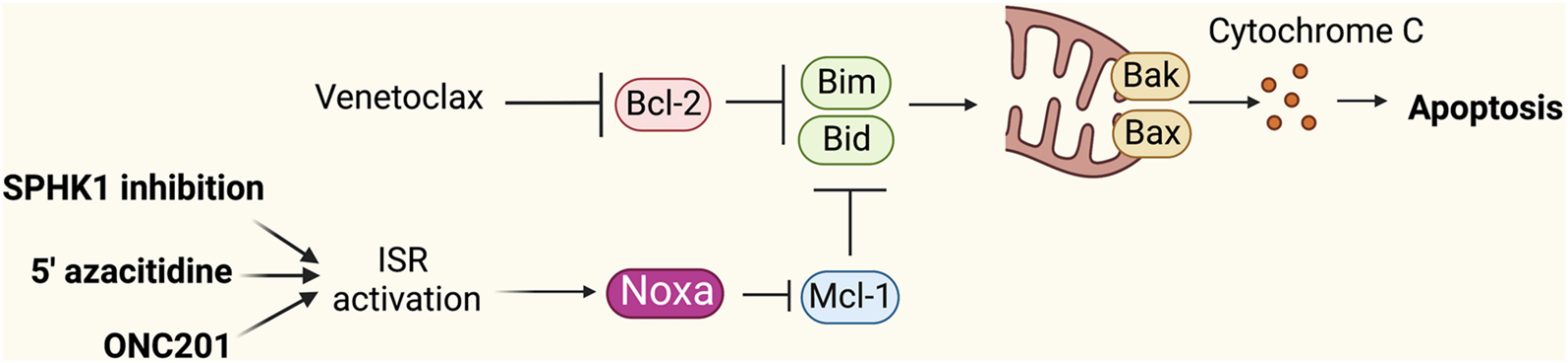
ISR activation may overcome resistance to the BH3-mimetic venetoclax. Mitochondrial apoptosis is regulated by the Bcl-2 family proteins which include the pro-survival proteins Bcl-2 and Mcl-1 and the pro-apoptotic proteins Bim, Bid, Noxa, Bak and Bax. Venetoclax inhibits the pro-survival protein Bcl-2 which allows Bim and Bid to activate Bak and Bax resulting in mitochondrial outer membrane permeabilization, release of cytochrome c (orange spheres) and induction of apoptosis. Resistance to venetoclax can arise through increased levels of the pro-survival protein Mcl-1 (not targeted by Venetoclax) which inhibits Bim and Bid to prevent apoptosis. Targeted inhibition of sphingosine kinase 1 (SPHK1), as well as the hypomethylating agent 5’azacitidine, and the dopamine receptor D2 (DRD2) agonist ONC201 all cause activation of the ISR to drive induction of NOXA (an Mcl-1 antagonist). This results in the inhibition of Mcl-1 to synergize with venetoclax and induce apoptosis in Venetoclax resistant cells. Created with BioRender.com

Epigenetic dysregulation is a common feature of a number of hematologic malignancies including AML, CML, chronic lymphocytic leukemia (CLL) and MDS. Accordingly, this has led to the discovery and clinical use of epigenetic modulating drugs in the management of these diseases [63]. One such drug is the hypomethylating agent 5’-azacitidine (5-Aza), which in AML has been shown to improve survival outcomes for elderly patients ineligible for intensive chemotherapy [64]. 5-Aza only elicits modest effects in AML when employed as a monotherapy (18% complete response rate; CRR [65]). However, combination of 5-Aza with the BH3 mimetic venetoclax, a selective antagonist of the anti-apoptotic Bcl-2 protein which also has modest effects in AML as a monotherapy (19% overall response rate [66]), results in impressive CRR of 76% [67]. Recently it was shown that 5-Aza induces apoptosis in AML via the upregulation of the pro-apoptotic protein NOXA in a TP53-independent manner (Fig. 5) [68]. Notably, 5-Aza was found to activate ISR signaling, as shown by an increased phosphorylation of eIF2α and induction of ATF4 (a known regulator of NOXA expression). Inhibition of eIF2α downregulated NOXA expression and led to a decrease in 5-Aza-induced AML cell death [68]. Thus, these studies provide insight into the mechanism underlying the enhanced effect of dual 5-Aza/venetoclax therapy and suggests that novel ISR-inducing agents used in combination with existing BH3-mimetics could be an effective strategy for the treatment of malignancies that are dependent on MCL1.

### Imipridones

Imipridones are a class of compound identified in a screen for small molecules capable of the inducing TNF-related apoptosis inducing ligand (TRAIL) in a TP53-independent manner in cancer cells [69]. The lead compound of this class, ONC201, has been shown to elicit anti-tumor effects in pre-clinical models across a range of solid tumors and hematologic malignancies, and is the subject of interest for multiple ongoing clinical trials [70]. The anti-neoplastic effects of ONC201 are attributed to induction of the extrinsic pathway of apoptosis via the induction of TRAIL and its receptor death receptor 5 (DR5) [71].

Mechanistically, ONC201 has primarily been characterized as a selective antagonist of dopamine receptor D2 (DRD2), a G-protein coupled receptor [72]. ONC201 was shown to induce the ISR via activation of PKR and HRI resulting in ATF4 and CHOP-dependent increases to DR5 levels [73]. In contrast, in both AML and mantle cell lymphoma (MCL) cell lines, ONC201 was shown to stimulate phosphorylation of GCN2 and eIF2α; however, eIF2α was found to be dispensable for ATF4 induction, suggesting that ONC201 stimulates a non-canonical ISR in hematologic malignancies [74]. Additionally, in contrast to solid tumors, ONC201 in hematologic malignancies did not stimulate TRAIL production but still induced apoptosis in a manner dependent on ATF4 upregulation [74].

Pre-clinical assessments of ONC201 in AML and MCL have led to further studies in panels of leukemia, lymphoma and MM cell lines, which have confirmed that the drug stimulates ISR activity as evidenced by the induction of ATF4 [75]. Interestingly, ONC201 has also been shown to downregulate the anti-apoptotic protein MCL1 in lymphoma cell lines and exhibited synergy with venetoclax against AML cell lines (Fig. 5) [74]. Given that ATF4 regulates the expression of NOXA, a known protein antagonist of MCL1, these findings further support the potential of modulating the ISR to therapeutically induce mitochondrial apoptosis in hematologic malignancies. This is of particular relevance given that resistance to BH3 mimetics is an emerging challenge to the efficacy of these drugs therapy of hematologic malignancies [76], thus sparking the need for novel agents that can circumvent these resistance mechanisms (Fig. 5).

Despite the promising pre-clinical efficacy of ONC201, a recent clinical trial combining the DRD2 antagonist thioridazine with intermediate dose cytarabine against relapsed/refractory AML demonstrated modest anti-leukemic activity with only one participant achieving a partial remission [77]. These findings cast doubt as to whether similar DRD2 targeting agents such as ONC201 will be therapeutically effective against hematologic malignancies; however, it must be noted that the pre-clinically determined optimal concentrations of thioridazine were not reached in this study due to dose-limiting toxicities associated with the drug [77]. Data obtained from the ongoing clinical trials of ONC201 in AML (NCT03932643, NCT02392572), MM (NCT02863991, NCT03492138) and non-Hodgkin’s lymphoma (NCT02420795) will potentially clarify the effectiveness of ONC201 as therapeutic approach against hematologic malignancies.

### Anthelmintics

Anthelmintics are a class of drugs commonly used in the treatment of parasitic infections but have garnered attention for potential drug-repurposing for cancer therapy [78]. In particular, a number of studies have examined the use of these agents against hematologic malignancies including AML [79, 80], ALL [81] and MM [82]. A recent study of the FDA approved anthelmintic drug pyrvinium pamoate in AML showed that it induced apoptotic cell death in FLT3-ITD harboring AML cell lines at low nanomolar concentrations via activation of the ISR, as shown by phosphorylation of eIF2α and upregulated expression of ATF4 [80]. Additionally, pyrvinium pamoate stimulated the increased transcription of a number of pro-apoptotic ATF4 target genes, including NOXA, PUMA and CHOP [80] further supporting the potential for modulation of the ISR as a therapeutic strategy for hematologic malignancies.

Atovaquone is another clinically available anthelmintic that stimulated the reduced in vitro viability of primary AML samples and lowered leukemic burden in murine models of MM and AML [79, 83]. Mechanistic studies of the anti-AML activity of atovaquone revealed that it induced the ISR, with increased phosphorylation of eIF2α and ATF4 protein levels while also increasing mRNA levels of numerous pro-apoptotic ATF4 target genes, including CHOP [79]. Furthermore, deletion of all four eIF2α kinases blocked atovaquone-stimulated phosphorylation of eIF2α, which was rescued by re-expression of PERK, and to some extent HRI, suggesting the involvement of these two eIF2α kinases in the activity of atovaquone [79]. Clarity on the mechanism of atovaquone in AML as well as its promising effects in pre-clinical studies has led to a Phase I trial currently examining its combination with standard induction chemotherapy regimens in younger AML patients (NCT03568994). Thus, the ISR inducing properties of drugs such as atovaquone and pyrvinium pamoate may be of significant value in a clinical setting, highlighting the potential for the advancement of other ISR modulating agents into clinical trials.

### Proteasome inhibitors

Cancer cells frequently exhibit elevated protein synthesis which makes them uniquely sensitive to proteasome inhibitors (PIs) that dysregulate protein homeostasis to cause proteotoxicity and cell death. In the context of hematologic malignancies, numerous studies have shown the PERK signaling pathway, at the junction of both the ISR and UPR, to play an important role in the effects of PIs. For instance, bortezomib is a PI approved for the treatment of MM and MCL and in MM cell lines bortezomib has been shown to induce increased levels of phospho-eIF2α, ATF4 and the expression of the pro-apoptotic ATF4 target gene CHOP [84]. Other PIs carfilzomib and marizomib elicited similar effects on eIF2α, ATF4 and CHOP in MM cells [85, 86].

These findings might suggest that ISR signaling is involved in PI-induced MM cell death. However, in MM cells PIs induce ISR/ATF4-driven expression of the pro-survival protein MCL1, and knockdown of ATF4 sensitizes MM cells to bortezomib, indicating a potential adaptive response triggered by bortezomib [87]. This, along with the effects of the ISR on suppressing global protein synthesis to reduce the unfolded protein load in MM cells, suggests the possibility of ISR signaling mediating resistance to PIs. Indeed, studies have suggested the potential of PERK inhibitors in MM therapy [88]. Never-the-less, together, these findings point towards a critical role for the ISR in modulating the effects of PIs and may provide complementary therapeutic strategies in the treatment of PI sensitive hematologic malignancies.

### Future directions: ISR activators as potential therapeutics in hematologic malignancies

Modulating ISR signaling represents a unique opportunity to exploit for the development of new anti-cancer therapies. Currently ISR induction can only be achieved by activating the upstream kinases or inhibiting the downstream phosphatases of eIF2α, as direct activators of ATF4 or eIF2α are not yet available.

Activation of GCN2 can be achieved by exploiting its natural regulation though amino acid deprivation. For example, GCN2 activation can be achieved with halofuginone, an alkaloid that binds to prolyl-tRNA synthetase to mimic the unavailability of proline [2], which has demonstrated anti-neoplastic properties against MM [89] and acute promyelocytic leukemia (APML) [90]. Similarly, pegylated arginine deiminase (ADI-PEG20), a modified enzyme which can be administered to degrade arginine and thus activate GCN2, has shown favorable results in clinical trials in AML patients in combination with low-dose cytarabine [91], although is ineffective against relapsed/refractory patients [92]. However, as noted earlier, inhibition of GCN2 sensitizes both ALL and AML cell lines to treatment with L-asparaginase [32], thus, indicating the complexity of the anti-leukemic roles of GCN2.

Notably, a number of direct small molecule activators of the eIF2α kinases have been recently developed which warrant further investigation as potential therapeutics in hematologic malignancies. Indeed, the small molecule PERK activator CCT020312 has been shown to overcome venetoclax resistance in AML [93]. As discussed above, HRI and PKR activation using ONC201 has been tested clinically in a range of hematologic malignancies. The small molecule HRI activator BTdCPU has also been shown to induce cell death in B-ALL [40] and numerous solid cancer cell lines [94]. Similarly, the small molecule PKR activator BEPP exhibits anti-cancer properties in both pancreatic [95] and lung cancer models [96] and may offer an exciting opportunity for further pre-clinical evaluation in hematologic malignancies.

We have recently shown that the sphingolipid ceramide activates PKR to induce cell death in AML and synergizes with the Bcl-2 inhibitor venetoclax in vitro and in patient derived xenografts models of AML [21]. In this study, ceramide levels were enhanced via inhibition of sphingosine kinase 1 (SPHK1) [21], with ceramide subsequently directly binding to and activating PKR to drive eIF2α phosphorylation and induction of ATF4. This resulted in the upregulation of the BH3-only protein NOXA and subsequent binding and downregulation of the pro-survival factor MCL1 to induce apoptotic cell death (Fig. 5). As MCL1 is known to mediate resistance to Bcl-2 inhibition in AML, combinational approaches of venetoclax with SPHK1 inhibitors induced synergistic AML cell death.

Another approach to enhancing eIF2α phosphorylation and ISR signaling is to employ small molecule inhibitors of the eIF2α protein phosphatase complex (Fig. 1). Salubrinal, guanabenz and its derivative Sephin1 have been shown to inhibit dephosphorylation of eIF2α by blocking GADD34 binding to the catalytic subunit of PP1 [97, 98]. Notably these agents have been shown to enhance ISR signaling and cell death when used alone or in combinational approaches in CML [99]. Nelfinavir (a HIV protease inhibitor) is the most well characterized PP1 complex inhibitor that induces downregulation of CReP by an undefined mechanism leading to reduced PP1c/CReP binding and enhanced eIF2α phosphorylation [100]. Nelfinavir has been extensively studied preclinically and in clinical trials in numerous hematologic malignancies with varied results and efficacies [101]. The potential clinical application of Nelfinavir is highlighted by a phase II clinical trial in refractory proteosome-resistant MM achieving an overall response rate (ORR) of > 65%, the highest ORR observed in resistant phase II/III trials to date [102]. Nelfinavir represents a striking example of new avenues to define paradigms of harnessing the activation of the ISR and warrants investigation for the treatment of hematologic malignancies.

The study of direct ISR modulating agents remains a relatively new field of research. However, the ISR has extensive roles in the biology of hematologic malignancies and in the mechanism of numerous targeted drugs. Thus, the continued development of selective ISR modulating agents merits further exploration and may yield novel agents to add to the arsenal of targeted therapies for hematologic malignancies.

## Conclusions

**Fig. 6:**
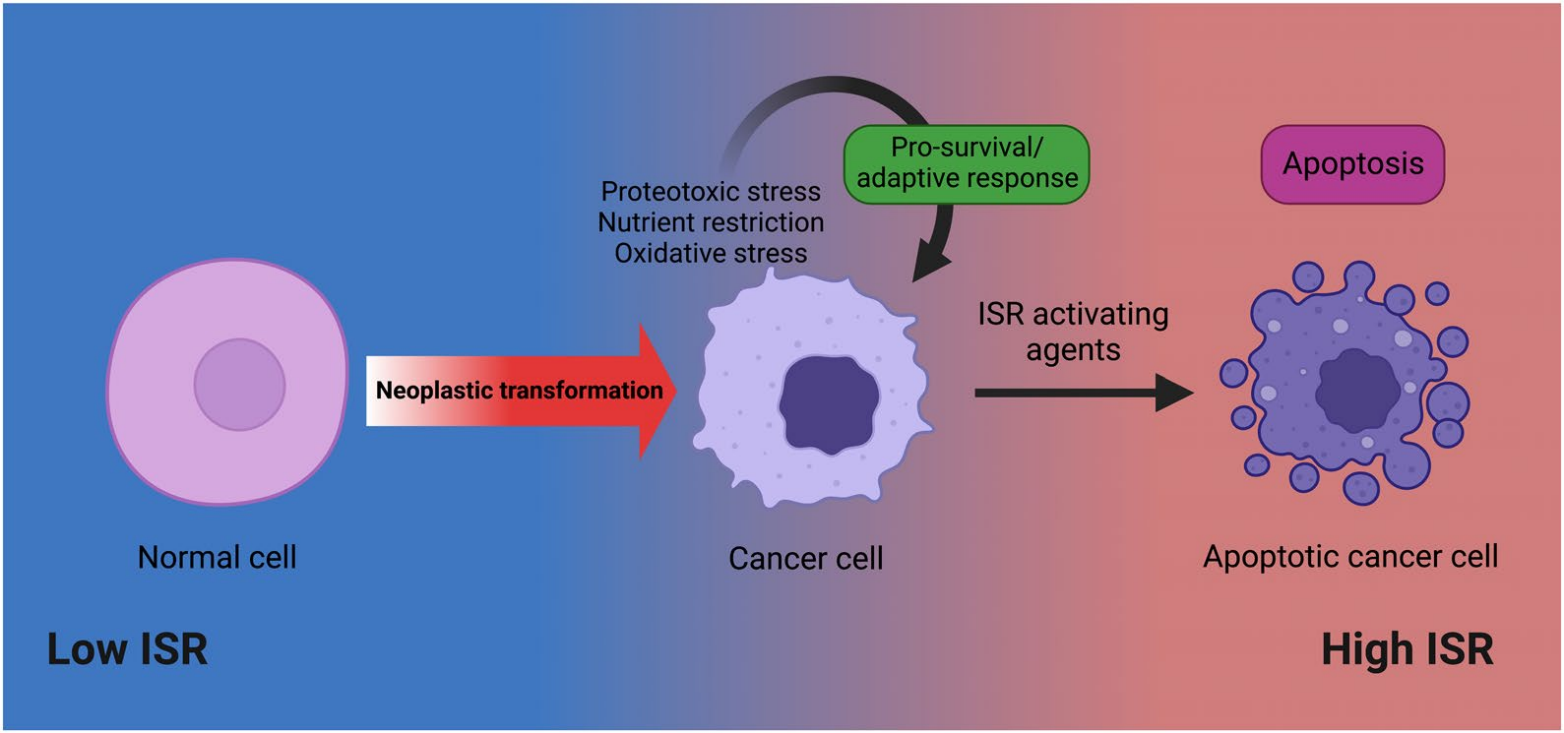
Differing stress stimuli influence the outcomes of ISR signaling. During homeostasis, normal cells (shown in pink) experience minimal stress and exhibit low ISR signaling. Normal cells can undergo neoplastic transformation to form cancer cells (light purple) which as a consequence of their excessive proliferation and altered metabolism are subject to stimuli such as proteotoxic stress, nutrient restriction and oxidative stress. These chronic stress stimuli trigger a low to moderate level of ISR signaling which can result in a pro-survival feedback response to facilitate cancer cell adaptation to stress. Extrinsic agents that activate the ISR cause a high degree of acute ISR signaling causing the cancer cell to undergo apoptosis. Created with BioRender.com

The ISR has emerged as an important signaling pathway implicated in the biology of numerous hematologic malignancies and its role in the pathogenesis of cancers is an area of growing interest. Recent years have seen the rapid development and implementation of targeted therapies for the treatment of hematologic malignancies and subsequent mechanistic studies of these agents have revealed a pattern of ISR modulation across multiple malignancies. The ISR elicits diverse outcomes on cell fate (Fig. 6), thus, explaining the capacity of targeted therapies and the ISR activating agents (discussed above) to exert their effects either through overstimulation of the ISR or blockade of the adaptive function of this pathway. The involvement of the ISR in targeted therapies suggests that future work should investigate the modulation of the ISR as a means of enhancing the efficacy of molecularly targeted agents. This concept of maximizing the ISR may also be useful in the design of novel combinations of targeted therapies. Given the role that the ISR may have in promoting drug resistance, these findings also support the ISR as a novel, targetable pathway that may be used to overcome resistance to clinically used therapies. For many hematologic malignancies, monotherapy with targeted agents is insufficient to elicit favorable outcomes, and thus efforts are increasing to identify rational drug combinations for clinical use. Therefore, targeting the ISR presents a novel and exciting paradigm for the treatment of hematologic malignancies.

## Data Availability

Not applicable.

## Abbreviations

ISR: Integrated stress response
PP1: Protein phosphatase 1
PP1c: Protein phosphatase 1 catalytic subunit
eIF2α: Eukaryotic initiation factor 2α
ATF4: Activating transcription factor 4
PERK: Protein kinase R-like endoplasmic reticulum kinase
ER: Endoplasmic reticulum
SP: Signal peptide
LD: Luminal domain
KD: Kinase domain
dsRBD: Double stranded RNA binding domain
PKD: Pseudokinase domain
HisRS: Histidyl-tRNA synthetase domain
CTD: C-terminal ribosome binding/dimerization domain
NTD: N-terminal heme binding domain
KD1: Kinase domain 1
KI: Kinase insert
KD2: Kinase domain 2
GRP78: 78 KDa glucose-regulated protein
MM: Multiple myeloma
CML: Chronic myeloid leukemia
PKR: Protein kinase R
dsRNA: Double-stranded RNA
ALL: Acute lymphoblastic leukemia
AML: Acute myeloid leukemia
MDS: Myelodysplastic syndrome
GCN2: General control non-derepressible kinase 2
RWD: Ring-finger proteins, WD-repeat containing proteins and DEAD-like helicases
HisRS: Histidyl-tRNA synthetase (HisRS)
CTD: C-terminal ribosome binding/dimerization domain
HRI: Heme regulated inhibitor
B-ALL: B-cell ALL
TC: Ternary complex
Met-tRNA_i_: Methionyl-initiator tRNA
PIC: Pre-initiation complex
MMPs: Matrix metalloproteinases
bZIP: Basic leucine zipper
PRMT5: Protein arginine methyltransferase 5
CDS: Coding sequence
uORF: Upstream open reading frames
βTrCP: β-Transducin repeat-containing protein
C/EBP: CCAAT box/enhancer binding protein
CHOP: C/EBP homologous protein
DLBCL: Diffuse large b-cell lymphoma
ROS: Reactive oxygen species
FLT3-ITD: Fms-like tyrosine kinase 3-internal tandem duplication
CLL: Chronic lymphocytic leukemia
5-Aza: 5’-Azacitidine
TRAIL: TNF-related apoptosis inducing ligand
DR5: Death receptor 5
DRD2: Dopamine receptor D2
MCL: Mantle cell lymphoma.
PI: Proteasome inhibitors
APML: Acute promyelocytic leukemia
ADI-PEG20: Pegylated arginine deiminase
SPHK1: Sphingosine kinase 1

## References

[1] Shimada A (2019). Hematological malignancies and molecular targeting therapy. Eur J Pharmacol.

[2] Pakos-Zebrucka K, Koryga I, Mnich K, Ljujic M, Samali A, Gorman AM (2016). The integrated stress response. EMBO Rep.

[3] Hinnebusch AG, Ivanov IP, Sonenberg N (2016). Translational control by 5′-untranslated regions of eukaryotic mRNAs. Science.

[4] Wortel IMN, van der Meer LT, Kilberg MS, van Leeuwen FN (2017). Surviving stress: modulation of ATF4-mediated stress responses in normal and malignant cells. Trends Endocrinol Metab.

[5] Cl J, Oyadomari S, Novoa I, Lu P, Zhang Y, Harding HP (2003). Inhibition of a constitutive translation initiation factor 2α phosphatase, CReP, promotes survival of stressed cells. J Cell Biol.

[6] Novoa I, Zeng H, Harding HP, Ron D (2001). Feedback inhibition of the unfolded protein response by GADD34-mediated dephosphorylation of eIF2α. J Cell Biol.

[7] Donnelly N, Gorman AM, Gupta S, Samali A (2013). The eIF2alpha kinases: their structures and functions. Cell Mol Life Sci.

[8] Bennett MK, Wallington-Beddoe CT, Pitson SM (2019). Sphingolipids and the unfolded protein response. Biochim Biophys Acta.

[9] Wang P, Li J, Tao J, Sha B (2018). The luminal domain of the ER stress sensor protein PERK binds misfolded proteins and thereby triggers PERK oligomerization. J Biol Chem.

[10] Shi Y, Vattem KM, Sood R, An J, Liang J, Stramm L (1998). Identification and characterization of pancreatic eukaryotic initiation factor 2 α-subunit kinase, PEK, involved in translational control. Mol Cell Biol.

[11] Michallet A-S, Mondiere P, Taillardet M, Leverrier Y, Genestier L, Defrance T (2011). Compromising the unfolded protein response induces autophagy-mediated cell death in multiple myeloma cells. PLoS ONE.

[12] Kusio-Kobialka M, Podszywalow-Bartnicka P, Peidis P, Glodkowska-Mrowka E, Wolanin K, Leszak G (2012). The PERK-eIF2α phosphorylation arm is a pro-survival pathway of BCR-ABL signaling and confers resistance to imatinib treatment in chronic myeloid leukemia cells. Cell Cycle.

[13] Nikesitch N, Lee JM, Ling S, Roberts TL (2018). Endoplasmic reticulum stress in the development of multiple myeloma and drug resistance. Clin Transl Immunol.

[14] Hart LS, Cunningham JT, Datta T, Dey S, Tameire F, Lehman SL (2012). ER stress–mediated autophagy promotes Myc-dependent transformation and tumor growth. J Clin Invest.

[15] Wang P, Han L, Yu M, Cao Z, Li X, Shao Y (2021). The prognostic value of PERK in cancer and its relationship with immune cell infiltration. Front Mol Biosci.

[16] Jeffrey IW, Kadereit S, Meurs EF, Metzger T, Bachmann M, Schwemmle M (1995). Nuclear localization of the interferon-inducible protein kinase PKR in human cells and transfected mouse cells. Exp Cell Res.

[17] Garcia-Ortega M, Lopez G, Jimenez G, Garcia-Garcia J, Conde V, Boulaiz H (2017). Clinical and therapeutic potential of protein kinase PKR in cancer and metabolism. Expert Rev Mol Med.

[18] Kim Y, Park J, Kim S, Kim M, Kang M-G, Kwak C (2018). PKR senses nuclear and mitochondrial signals by interacting with endogenous double-stranded RNAs. Mol Cell.

[19] Nakamura T, Furuhashi M, Li P, Cao H, Tuncman G, Sonenberg N (2010). Double-stranded RNA-dependent protein kinase links pathogen sensing with stress and metabolic homeostasis. Cell.

[20] Patel CV, Handy I, Goldsmith T, Patel RC (2000). PACT, a stress-modulated cellular activator of interferon-induced double-stranded RNA-activated protein kinase. PKR J Biol Chem.

[21] Lewis AC, Pope VS, Tea MN, Li M, Nwosu GO, Nguyen TM (2022). Ceramide-induced integrated stress response overcomes Bcl-2 inhibitor resistance in acute myeloid leukemia. Blood.

[22] Chukwurah E, Farabaugh KT, Guan BJ, Ramakrishnan P, Hatzoglou M (2021). A tale of two proteins: PACT and PKR and their roles in inflammation. FEBS J.

[23] Basu S, Panayiotidis P, Hart S, He L, Man A, Hoffbrand A (1997). Role of double-stranded RNA-activated protein kinase in human hematological malignancies. Cancer Res.

[24] Cheng X, Byrne M, Brown KD, Konopleva MY, Kornblau SM, Bennett RL (2015). PKR inhibits the DNA damage response, and is associated with poor survival in AML and accelerated leukemia in NHD13 mice. Blood.

[25] Blalock WL, Grimaldi C, Fala F, Follo M, Horn S, Basecke J (2009). PKR activity is required for acute leukemic cell maintenance and growth: a role for PKR-mediated phosphatase activity to regulate GSK-3 phosphorylation. J Cell Physiol.

[26] Liu X, Bennett RL, Cheng X, Byrne M, Reinhard MK, May WS (2013). PKR regulates proliferation, differentiation, and survival of murine hematopoietic stem/progenitor cells. Blood.

[27] Follo MY, Finelli C, Mongiorgi S, Clissa C, Bosi C, Martinelli G (2008). PKR is activated in MDS patients and its subcellular localization depends on disease severity. Leukemia.

[28] Masson GR (2019). Towards a model of GCN2 activation. Biochem Soc Trans.

[29] Castilho BA, Shanmugam R, Silva RC, Ramesh R, Himme BM, Sattlegger E (2014). Keeping the eIF2 alpha kinase Gcn2 in check. Biochim Biophys Acta.

[30] Wu CC-C, Peterson A, Zinshteyn B, Regot S, Green R. Ribosome collisions trigger general stress responses to regulate cell fate. Cell. 2020;182(2):404–416.e414. 10.1016/j.cell.2020.06.006 PMC7384957 32610081

[31] Tabe Y, Lorenzi PL, Konopleva M (2019). Amino acid metabolism in hematologic malignancies and the era of targeted therapy. Blood.

[32] Nakamura A, Nambu T, Ebara S, Hasegawa Y, Toyoshima K, Tsuchiya Y (2018). Inhibition of GCN2 sensitizes ASNS-low cancer cells to asparaginase by disrupting the amino acid response. Proc Natl Acad Sci.

[33] Emadi A, Zokaee H, Sausville EA (2014). Asparaginase in the treatment of non-ALL hematologic malignancies. Cancer Chemother Pharmacol.

[34] Croucher DC, Richards LM, Tsofack SP, Waller D, Li Z, Wei EN (2021). Longitudinal single-cell analysis of a myeloma mouse model identifies subclonal molecular programs associated with progression. Nat Commun.

[35] Chen J-J, Zhang S (2019). Heme-regulated eIF2α kinase in erythropoiesis and hemoglobinopathies. Blood.

[36] Han A-P, Yu C, Lu L, Fujiwara Y, Browne C, Chin G (2001). Heme-regulated eIF2α kinase (HRI) is required for translational regulation and survival of erythroid precursors in iron deficiency. EMBO J.

[37] Lu L, Han A-P, Chen J-J (2001). Translation initiation control by heme-regulated eukaryotic initiation factor 2α kinase in erythroid cells under cytoplasmic stresses. Mol Cell Biol.

[38] Guo X, Aviles G, Liu Y, Tian R, Unger BA, Lin YT (2020). Mitochondrial stress is relayed to the cytosol by an OMA1-DELE1-HRI pathway. Nature.

[39] Burwick N, Zhang MY, de la Puente P, Azab AK, Hyun TS, Ruiz-Gutierrez M (2017). The eIF2-alpha kinase HRI is a novel therapeutic target in multiple myeloma. Leukemia Res.

[40] Smith KH, Budhraja A, Lynch J, Roberts K, Panetta JC, Connelly JP (2020). The heme-regulated inhibitor pathway modulates susceptibility of poor prognosis B-lineage acute leukemia to BH3-mimetics. Mol Cancer Res.

[41] Jackson RJ, Hellen CUT, Pestova TV (2010). The mechanism of eukaryotic translation initiation and principles of its regulation. Nat Rev Mol Cell Biol.

[42] Costa-Mattioli M, Walter P (2020). The integrated stress response: From mechanism to disease. Science.

[43] Pavitt GD (2018). Regulation of translation initiation factor eIF2B at the hub of the integrated stress response. Wiley Interdiscip Rev RNA.

[44] Podszywalow-Bartnicka P, Cmoch A, Wolczyk M, Bugajski L, Tkaczyk M, Dadlez M (2016). Increased phosphorylation of eIF2α in chronic myeloid leukemia cells stimulates secretion of matrix modifying enzymes. Oncotarget.

[45] Kasai S, Yamazaki H, Tanji K, Engler MJ, Matsumiya T, Itoh K (2018). Role of the ISR-ATF4 pathway and its cross talk with Nrf2 in mitochondrial quality control. J Clin Biochem Nutr.

[46] Dey S, Baird TD, Zhou D, Palam LR, Spandau DF, Wek RC (2010). Both transcriptional regulation and translational control of ATF4 are central to the integrated stress response. J Biol Chem.

[47] Martina JA, Diab HI, Brady OA, Puertollano R (2016). TFEB and TFE 3 are novel components of the integrated stress response. EMBO J.

[48] Szewczyk MM, Luciani GM, Vu V, Murison A, Dilworth D, Barghout SH (2022). PRMT5 regulates ATF4 transcript splicing and oxidative stress response. Redox Biol.

[49] Vattem KM, Wek RC (2004). Reinitiation involving upstream ORFs regulates ATF4 mRNA translation in mammalian cells. Proc Natl Acad Sci.

[50] Lu PD, Harding HP, Ron D (2004). Translation reinitiation at alternative open reading frames regulates gene expression in an integrated stress response. J Cell Biol.

[51] Lassot I, Estrabaud E, Emiliani S, Benkirane M, Benarous R, Margottin-Goguet F (2005). p300 modulates ATF4 stability and transcriptional activity independently of its acetyltransferase domain. J Biol Chem.

[52] Köditz J, Nesper J, Wottawa M, Stiehl DP, Camenisch G, Franke C (2007). Oxygen-dependent ATF-4 stability is mediated by the PHD3 oxygen sensor. Blood.

[53] Karpinski BA, Morle GD, Huggenvik J, Uhler MD, Leiden JM (1992). Molecular cloning of human CREB-2: an ATF/CREB transcription factor that can negatively regulate transcription from the cAMP response element. Proc Natl Acad Sci.

[54] Lassot I, Ségéral E, Berlioz-Torrent C, Durand H, Groussin L, Hai T (2001). ATF4 degradation relies on a phosphorylation-dependent interaction with the SCFβTrCPUbiquitin ligase. Mol Cell Biol.

[55] Podust LM, Krezel AM, Kim Y (2001). Crystal structure of the CCAAT box/enhancer-binding protein β activating transcription factor-4 basic leucine zipper heterodimer in the absence of DNA. J Biol Chem.

[56] Han J, Back SH, Hur J, Lin Y-H, Gildersleeve R, Shan J (2013). ER-stress-induced transcriptional regulation increases protein synthesis leading to cell death. Nat Cell Biol.

[57] Singleton DC, Harris AL (2012). Targeting the ATF4 pathway in cancer therapy. Expert Opin Ther Targets.

[58] Li M, Teater MR, Hong JY, Park NR, Duy C, Shen H (2022). Translational activation of ATF4 through mitochondrial anaplerotic metabolic pathways is required for DLBCL growth and survival. Blood Cancer Discov.

[59] Heydt Q, Larrue C, Saland E, Bertoli S, Sarry J, Besson A (2018). Oncogenic FLT3-ITD supports autophagy via ATF4 in acute myeloid leukemia. Oncogene.

[60] Williams MS, Somervaille TC (2020). Dynamic induction of drug resistance through a stress-responsive enhancer in acute myeloid leukemia. Mol Cell Oncol.

[61] van Galen P, Mbong N, Kreso A, Schoof EM, Wagenblast E, Ng SWK (2018). Integrated stress response activity marks stem cells in normal hematopoiesis and leukemia. Cell Rep.

[62] Matsumura T, Nakamura-Ishizu A, Muddineni SSNA, Tan DQ, Wang CQ, Tokunaga K (2020). Hematopoietic stem cells acquire survival advantage by loss of RUNX1 methylation identified in familial leukemia. Blood.

[63] Gardin C, Dombret H (2017). Hypomethylating agents as a therapy for AML. Curr Hematol Malig Rep.

[64] Dombret H, Seymour JF, Butrym A, Wierzbowska A, Selleslag D, Jang JH (2015). International phase 3 study of azacitidine vs conventional care regimens in older patients with newly diagnosed AML with > 30% blasts. Blood.

[65] Fenaux P, Mufti GJ, Hellström-Lindberg E, Santini V, Gattermann N, Germing U (2010). Azacitidine prolongs overall survival compared with conventional care regimens in elderly patients with low bone marrow blast count acute myeloid leukemia. J Clin Oncol.

[66] Konopleva M, Pollyea DA, Potluri J, Chyla B, Hogdal L, Busman T (2016). Efficacy and biological correlates of response in a phase II study of venetoclax monotherapy in patients with acute myelogenous leukemia. Cancer Discov.

[67] DiNardo CD, Pratz K, Pullarkat V, Jonas BA, Arellano M, Becker PS (2019). Venetoclax combined with decitabine or azacitidine in treatment-naive, elderly patients with acute myeloid leukemia. Blood.

[68] Jin S, Cojocari D, Purkal JJ, Popovic R, Talaty NN, Xiao Y (2020). 5-Azacitidine induces NOXA to prime AML cells for venetoclax-mediated apoptosis. Clin Cancer Res.

[69] Allen JE, Krigsfeld G, Patel L, Mayes PA, Dicker DT, Wu GS (2015). Identification of TRAIL-inducing compounds highlights small molecule ONC201/TIC10 as a unique anti-cancer agent that activates the TRAIL pathway. Mol Cancer.

[70] Prabhu VV, Morrow S, Kawakibi AR, Zhou L, Ralff M, Ray J (2020). ONC201 and imipridones: anti-cancer compounds with clinical efficacy. Neoplasia.

[71] Allen JE, Kline CLB, Prabhu VV, Wagner J, Ishizawa J, Madhukar N (2016). Discovery and clinical introduction of first-in-class imipridone ONC201. Oncotarget.

[72] Kline CLB, Ralff MD, Lulla AR, Wagner JM, Abbosh PH, Dicker DT (2018). Role of dopamine receptors in the anticancer activity of ONC201. Neoplasia.

[73] Kline CLB, Van den Heuvel APJ, Allen JE, Prabhu VV, Dicker DT, El-Deiry WS (2016). ONC201 kills solid tumor cells by triggering an integrated stress response dependent on ATF4 activation by specific eIF2α kinases. Sci Signal.

[74] Ishizawa J, Kojima K, Chachad D, Ruvolo P, Ruvolo V, Jacamo RO (2016). ATF4 induction through an atypical integrated stress response to ONC201 triggers p53-independent apoptosis in hematological malignancies. Sci Signal.

[75] Prabhu VV, Talekar MK, Lulla AR, Kline CLB, Zhou L, Hall J (2018). Single agent and synergistic combinatorial efficacy of first-in-class small molecule imipridone ONC201 in hematological malignancies. Cell Cycle.

[76] DiNardo CD, Tiong IS, Quaglieri A, MacRaild S, Loghavi S, Brown FC (2020). Molecular patterns of response and treatment failure after frontline venetoclax combinations in older patients with AML. Blood.

[77] Aslostovar L, Boyd AL, Almakadi M, Collins TJ, Leong DP, Tirona RG (2018). A phase 1 trial evaluating thioridazine in combination with cytarabine in patients with acute myeloid leukemia. Blood Adv.

[78] Laudisi F, Marônek M, Di Grazia A, Monteleone G, Stolfi C (2020). Repositioning of anthelmintic drugs for the treatment of cancers of the digestive system. Int J Mol Sci.

[79] Stevens AM, Xiang M, Heppler LN, Tošić I, Jiang K, Munoz JO (2019). Atovaquone is active against AML by upregulating the integrated stress pathway and suppressing oxidative phosphorylation. Blood Adv.

[80] Fu Y-H, Tseng C-Y, Lu J-W, Lu W-H, Lan P-Q, Chen C-Y (2021). Deciphering the role of pyrvinium pamoate in the generation of integrated stress response and modulation of mitochondrial function in myeloid leukemia cells through transcriptome analysis. Biomedicines.

[81] Mezzatesta C, Abduli L, Guinot A, Eckert C, Schewe D, Zaliova M (2020). Repurposing anthelmintic agents to eradicate resistant leukemia. Blood Cancer J.

[82] Xu F, Zhu Y, Lu Y, Yu Z, Zhong J, Li Y (2018). Anthelmintic pyrvinium pamoate blocks Wnt/β-catenin and induces apoptosis in multiple myeloma cells. Oncol Lett.

[83] Xiang M, Kim H, Ho VT, Walker SR, Bar-Natan M, Anahtar M (2016). Gene expression–based discovery of atovaquone as a STAT3 inhibitor and anticancer agent. Blood.

[84] Obeng EA, Carlson LM, Gutman DM, Harrington WJ, Lee KP, Boise LH (2006). Proteasome inhibitors induce a terminal unfolded protein response in multiple myeloma cells. Blood.

[85] Chauhan D, Singh A, Brahmandam M, Podar K, Hideshima T, Richardson P (2008). Combination of proteasome inhibitors bortezomib and NPI-0052 trigger in vivo synergistic cytotoxicity in multiple myeloma. Blood.

[86] Bennett MK, Li M, Tea MN, Pitman MR, Toubia J, Wang PP-S (2022). Resensitising proteasome inhibitor-resistant myeloma with sphingosine kinase 2 inhibition. Neoplasia.

[87] Hu J, Dang N, Menu E, De Bryune E, Xu D, Van Camp B (2012). Activation of ATF4 mediates unwanted Mcl-1 accumulation by proteasome inhibition. Blood.

[88] Atkins C, Liu Q, Minthorn E, Zhang S-Y, Figueroa DJ, Moss K (2013). Characterization of a novel PERK kinase inhibitor with antitumor and antiangiogenic activity. Cancer Res.

[89] Leiba M, Jakubikova J, Klippel S, Mitsiades CS, Hideshima T, Tai YT (2012). Halofuginone inhibits multiple myeloma growth in vitro and in vivo and enhances cytotoxicity of conventional and novel agents. Br J Haematol.

[90] Assis PA, Figueiredo-Pontes D, Lorena L, Lima ASG, Leão V, Cândido LA (2015). Halofuginone inhibits phosphorylation of SMAD-2 reducing angiogenesis and leukemia burden in an acute promyelocytic leukemia mouse model. J Exp Clin Cancer Res.

[91] Tsai HJ, Hsiao HH, Hsu YT, Liu YC, Kao HW, Liu TC (2021). Phase I study of ADI-PEG20 plus low-dose cytarabine for the treatment of acute myeloid leukemia. Cancer Med.

[92] Tsai H-J, Jiang SS, Hung W-C, Borthakur G, Lin S-F, Pemmaraju N (2017). A phase II study of arginine deiminase (ADI-PEG20) in relapsed/refractory or poor-risk acute myeloid leukemia patients. Sci Rep.

[93] Sharon D, Cathelin S, Mirali S, Di Trani JM, Yanofsky DJ, Keon KA (2019). Inhibition of mitochondrial translation overcomes venetoclax resistance in AML through activation of the integrated stress response. Sci Transl Med..

[94] Chen T, Ozel D, Qiao Y, Harbinski F, Chen L, Denoyelle S (2011). Chemical genetics identify eIF2α kinase heme-regulated inhibitor as an anticancer target. Nat Chem Biol.

[95] Song Y, Wan X, Gao L, Pan Y, Xie W, Wang H (2015). Activated PKR inhibits pancreatic β-cell proliferation through sumoylation-dependent stabilization of P53. Mol Immunol.

[96] Hu W, Hofstetter W, Wei X, Guo W, Zhou Y, Pataer A (2009). Double-stranded RNA-dependent protein kinase-dependent apoptosis induction by a novel small compound. J Pharmacol Exp Ther.

[97] Boyce M, Bryant KF, Jousse C, Long K, Harding HP, Scheuner D (2005). A selective inhibitor of eIF2α dephosphorylation protects cells from ER stress. Science.

[98] Das I, Krzyzosiak A, Schneider K, Wrabetz L, D’Antonio M, Barry N (2015). Preventing proteostasis diseases by selective inhibition of a phosphatase regulatory subunit. Science.

[99] Drexler HC (2009). Synergistic apoptosis induction in leukemic cells by the phosphatase inhibitor salubrinal and proteasome inhibitors. PLoS ONE.

[100] De Gassart A, Bujisic B, Zaffalon L, Decosterd LA, Di Micco A, Frera G (2016). An inhibitor of HIV-1 protease modulates constitutive eIF2α dephosphorylation to trigger a specific integrated stress response. Proc Natl Acad Sci.

[101] Allegra A, Innao V, Allegra A, Pulvirenti N, Pugliese M, Musolino C (2020). Antitumorigenic action of nelfinavir: Effects on multiple myeloma and hematologic malignancies. Oncol Rep.

[102] Driessen C, Müller R, Novak U, Cantoni N, Betticher D, Mach N (2018). Promising activity of nelfinavir-bortezomib-dexamethasone in proteasome inhibitor–refractory multiple myeloma. Blood.

